# Investigating the Causes of Medication Errors and Strategies to Prevention of Them from Nurses and Nursing Student Viewpoint

**DOI:** 10.5539/gjhs.v8n8p220

**Published:** 2015-12-17

**Authors:** Enam Alhagh Charkhat Gorgich, Sanam Barfroshan, Gholamreza Ghoreishi, Maryam Yaghoobi

**Affiliations:** 1 Student Scientific Research Center, Pregnancy Health Research Center, Zahedan University of Medical Sciences, Zahedan, Iran; 2 Pregnancy Health Research Center, Zahedan University of Medical Sciences, Zahedan, Iran

**Keywords:** medication errors, nurse, nursing students, prevention, strategies, viewpoint

## Abstract

**Introduction and Aim:**

Medication errors as a serious problem in world and one of the most common medical errors that threaten patient safety and may lead to even death of them. The purpose of this study was to investigate the causes of medication errors and strategies to prevention of them from nurses and nursing student viewpoint.

**Materials & Methods:**

This cross-sectional descriptive study was conducted on 327 nursing staff of khatam-al-anbia hospital and 62 intern nursing students in nursing and midwifery school of Zahedan, Iran, enrolled through the availability sampling in 2015. The data were collected by the valid and reliable questionnaire. To analyze the data, descriptive statistics, T-test and ANOVA were applied by use of SPSS16 software.

**Findings:**

The results showed that the most common causes of medications errors in nursing were tiredness due increased workload (97.8%), and in nursing students were drug calculation, (77.4%). The most important way for prevention in nurses and nursing student opinion, was reducing the work pressure by increasing the personnel, proportional to the number and condition of patients and also creating a unit as medication calculation. Also there was a significant relationship between the type of ward and the mean of medication errors in two groups.

**Conclusion:**

Based on the results it is recommended that nurse-managers resolve the human resources problem, provide workshops and in-service education about preparing medications, side-effects of drugs and pharmacological knowledge. Using electronic medications cards is a measure which reduces medications errors.

## 1. Introduction

Some health problems in today’s world including obesity, cardiovasular diseases, cancer, addiction, diabetes are accompanied with changes in life style and in turn, lead in taking drugs (Arbabisarjou, Robabi, 2015). Medication errors are the most common medical errors that can occur as inappropriate use of medicine in each one of medicine prescription stages for patients (Fontan & Maneglier, 2003; Hansen & Greene, 2006; Wolf & Hicks, 2006). Medical errors include prescribing the wrong medicine at any stage of the treatment process that is preventable (Woods & Doan-Johnson, 2002). Giving drug is one of the most important, complexes, yet most vital processes of nursing care and it needs the right knowledge and function of nurses. Medication errors can have undesirable consequences for patients such as: Increased length of hospitalization, increased costs of hospitalization, disability and distrust in the healthcare system, severe injury or even patient death (Webster & Anderson, 2002). Implementation of medication orders is an important part of the process of treatment and care of patients and it’s considered as a major component of nurse’s function, and in the meantime patient safety has a particular importance (Soozani & Bagheri, 2007). The first reports of medication errors were made in 1940 and they drew attention (Rahimi & Seyyed-Rasouli, 2004). Medication errors were classified as one of five medical error categories by (National Institute of General Medical Science) and (Institute of Medicine) (Mrayyan & Shishani, 2007). Studies have shown that almost in 44 to 98 thousand deaths due to medical errors, 7000 occurred due to medication errors. Costs resulting from medication errors were estimated between almost 6.1 to 6.5 billion dollars in United States of America (Stratton & Blegen, 2004; Grissinger & Kelly, 2005). A study conducted by Hughes and Ortiz in 2005 showed that 30% of patients affected by medical errors will die or will be disabled for more than six months (Hughes & Ortiz, 2005). In fact, nurses and nursing students in hospitals are people who are directly associated with giving drug to patients and they are known as people who may make the most medication errors (Clifton-koeppel, 2008). So that nurses spend 40 percent of their time on average in hospital for giving medicine to their patients (Demehin & Babalola, 2008). Today there are more than 20 thousand types of drugs in the world that all of them despite their therapeutic effects have Complications and their own instructions. So for that nurses and nursing students should have the necessary information about drugs to avoid potential dangers (Koohestani & Baghcheghi, 2008). Common medication errors in drug prescription include: making mistake in drugs concentration, not paying attention to the right time of using drug, over dosage of drug and not paying attention to the right way of using the drug (Woods & Doan-Johnson, 2002). Research has shown that the rate of medication errors by nurses and nursing students is high, however, the report of these errors by them is low (Blegen & Vaughn, 2004; Bennerm & Sheets, 2002; Kawamura, 2001). According to a study by McCarthy et al. rate of medication errors in nursing students was reported 48.5% and the most common type of medication errors was forgetting drug prescription (McCarthy & Kelly, 2000). In a study in 2004 by Balas and colleagues at the University of Pennsylvania conducted on 393 nurses, it was found that during the study, 30% of nurses surveyed, reported at least one error (Balas & Scott, 2004). Things such as lack of pharmacological information, wrong medication calculations, not regarding the defined protocols, similarity in shapes and pickings of drugs, similarity in drug names and physicians bad hand writing can cause medication errors (Carlton & Blegen, 2006). In theory all medication errors are preventable and almost a third of unwelcome drug events are preventable (Ghasemi & Valizadeh, 2009). Medication errors are multidimensional problems and for solving them we should find multilateral solutions. We can reduce the medication errors through risk management which it is a daily and continous program for dianosis and intervention. Risk mangement is a problem-centerd approach (Arbabisarjou, 2012). However determination of causes of medication errors is as the first step to prevent and control that. Therefore, this study aimed to investigate the causes of medication errors and their prevention strategies from the perspective of nurses and nursing students.

## 2. Materials and Methods

This study was a descriptive study that was conducted cross-sectional to investigate the causes of medication errors and their prevention strategies from the perspective of nurses and nursing students in 2015. Study population included all the nurses working in different wards of three specialty and subspecialty hospitals in Zahedan (ali-ebne-abitaleb, khatam-al-anbia, alzahra) and the nursing students of the nursing and midwifery school of Zahedan University of Medical Sciences. The sample volume consisted 327 nurses working in different wards of hospitals and 62 nursing students. Sampling was convenience and available (simple non-random). Inclusion criteria in this study for nurses was having at least one year of work experience in the current ward and having at least a bachelor’s degree in nursing and for students, senior nursing students were required to pass the course of pharmacology. Data collection tool was a questionnaire designed by the researcher, consisting of three parts. The questionnaire was used after confirming the validity and reliability. Face and content validity of the questionnaire was determined by ten educators, nurse educators and Statistics advisor. After collecting the opinions of these individuals small changes were given to the questionnaire. To verify reliability of questionnaire test-retest method was used, that the correlation coefficients between the two turns answering the questions in nursing and undergraduate students were 89.0 and 91.0 respectively and validity of the questionnaire was approved. The first part of the questionnaire was related to demographic information such as age, sex, working ward, working shift, work experience, type of employment and having a training course in the field of giving drug. The second part contained 22 items about the causes of medication errors in nurses and students viewpoint that they responded to them by YES and NO. The third part was about the ways of preventing medication errors in nurses and students viewpoint. Exclusion criteria in this study were lack of cooperation in filling out the questionnaires by the surveyed sections or uncompleted questionnaires. To respect the ethical considerations in research, first and above all the aim of the study and how to complete the questionnaire was described for surveyed groups and they were assured that the information will remain confidential and it’s not required to write the name. Inclusion to the study was voluntary based and it was up to oral satisfaction of sections.

Then with the permission of hospital and university administrators the questionnaires were distributed 16 times in different shifts(morning, afternoon, evening) between the sections and then after completing by them the questionnaires were collected. At the end for describing the data, descriptive statistics (frequency, mean and standard deviation) and analytical statistics (t-test and ANOVA) were used. All analysis was performed using SPSS for Windows (Version 16.0 SPSS inc., Evenston, Illinois). This study conducted after the adoption of the proposal in research Council and approving by the Ethics Committee of the Zahedan University of Medical Sciences. A significance level of 0.05 was adopted.

## 3. Findings

Based on the results, response rate was 88.66% for nurses and 100%. For students. All the nurses had bachelor’s degrees. Other demographic data of nurses and students have been presented in Table 1.

**Table 1:** Mean distribution, standard deviation and frequency of demographic information for surveyed nurses and students

Variable		Nurses	Nursing students
Age		**M±SD** 4/5±32	**M±SD** 21/5393/0±
		Frequency	
Sex	man	129	24
	woman	198	38
Ward	internal	118	13
	surgery	54	6
	emergency	56	7
	gynecology	37	-
	ICU	32	-
	pediatrics	30	10
Hospital	ALI-EBNE-ABITALEB	154	40
	KHATAM-AL-ANBIA	137	22
	AL-ZAHRA	36	-
Shift	Fixed	71	-
	Rotatory	256	
Employment	Official	103	-
	Contractual	87	
	Agreement	52	
	Projective	85	
having a training course in the field of giving drug	Have	152	-
	Don’t have	175
Job experience		M±SD 78/1±63/9	-

According to nurses view point, heavy workload, the large number of critically ill patients, doctor’s damaged and unreadable orders, the low ratio of nurses to patients and environmental conditions lead to distraction had the highest impact on medication errors in nursing. Other factors affecting the occurrence of medication errors are given in Table 2.

**Table 2:** Nurses views point on the influencing factors of medication errors in 2015

Cause(view point)	YES (%) number	NO (%) number
Fatigue due to high workload	(97.8)320	(2.1)7
the large number of critically ill patients	(89.9)294	(10)33
doctor’s damaged and unreadable orders	(88.6)290	(11.31)37
the low ratio of nurses to patients	(74)242	(25.9)85
environmental conditions lead to distraction (Noise, heavy traffic)	(69.7)228	(30.2)99
Large variety of drugs in Ward	(65.7)215	(34.2)112
Poor physical environment (light, temperature)	(58.4)191	(41.5)136
Accompanying of patient	(55.9)183	(44)144
Officials failure in emphasizing the importance of recording and reporting the medication errors	(54.4)178	(45.5)149
Poor communication between care team members	(52.2)171	(47.7)156
Blaming the staff by the administrator for reporting medication errors	(50.7)166	(49.2)161
Inappropriate relationship between manager and the staff	(43.4)142	(56.5)185
Improper location of medicinal shelves	(39.4)129	(60.5) 198
Blaming the staff by doctors for medication errors reported	(36.6)120	(63.3)207
Lack of the source of pharmacological information in the ward	(33.9)111	(66)216
Getting incompetence label due to medication errors reported	(29.9)98	(70)229
Blaming by colleagues for reporting medication errors	(28.4)93	(71.5)234
Inadequate drug label or packaging	(26.6)87	(73.3)240
The absence of recording and reporting system for errors	(22.3)73	(77.6)254
The lack of monitoring of the care process	(16.5)54	(83.4)273
Lack of awareness and Collective agreement of definition of medication errors	(14.3)47	(85.6)280
Working in an educational hospital	0	(100)327

According to independent t-test there wasn’t any significant relationship between gender and medication errors (p=0.08). Also, according to analysis of variance, there was a significant relationship between the working shift (p=0.012), type of employment (p=0.003) and type of ward (p=0.019) with the mean of medication errors occurred in nurses. So that the highest rate of medication errors in nurses with rotatory shift was reported in projective nurses and internal ward nurses. From the students view point, wrong drug calculation, lack of pharmacological information and doctor’s damaged and unreadable orders on medicine cards were reported as factors that have the greatest impact on medication errors. Other factors affecting the occurrence of medication errors from the student’s viewpoint are given in Table 3.

**Table 3:** Nursing students’ viewpoints about influencing factors of medication errors in 2015

Cause(view point)	YES (%) number	NO (%) number
Wrong medication calculation	(4/77)28	(6/22)14
Lack of pharmacological information	(8/75)47	(2/24)15
doctor’s damaged and unreadable orders on medicine cards	(6/72)45	(4/27)17
environmental conditions lead to distraction (Noise, heavy traffic)	(1/66)41	(9/33)21
Stress in emergency situations	(9/62)39	(1/37)23
Lack of attention to the dose of a drug on the medicine card	(3/61)38	(7/38)24
To do oral statements without checking the medicine card	(5/56)35	(5/43)27
Similarity in the name of drugs and reading the wrong name from the medicine card	(8/54)34	(2/45)28
Similarity in the drugs shape and lack of attention to the label of drugs	(2/53)33	(8/46)29
Different routine of wards in the concentration of infusion drugs	(6/51)32	(4/48)30
Failure to follow the process of infusion after injection	(50)31	(50)31
The use of acronyms instead of full name of drugs	(50)31	(50)31
Entering wrong drug in the medicine card	(4/48)30	(6/51)32
Similarity in the category of drugs	(9/41)26	(1/58)36
high workload	(3/40)25	(7/59)37
Not paying attention to the PRN order	(1/37)23	(9/62)39
Poor physical environment (light, temperature)	(5/35)22	(5/64)40
Poor clinical skills	(9/33)21	(1/66)41
Lack of familiarity with the drug injection equipments	(3/32)20	(7/67)42
Prescription of drugs without medical supervision	(6/30)19	(4/69)43
Not following-up the treatment methods	(29)18	(71)44
Working in an educational hospital	(2/24)15	(8/75)47

According to independent t-test there was a significant relationship between gender and medication errors among students (p=0.63). Also the ANOVA test showed a significant relationship between the ward and occurrence of medication errors among students (p=0.03). So that most errors were related to the internal ward. From nurse’s view point the most important way for prevention and controlling medication errors is to reduce working pressure and increase the number of staff proportional to the number of patients and in view point of nursing students it was to create a section as medication calculation to practice and improve the skills needed for calculating right dosage of the drugs. Other ways to prevent medication errors in separate surveyed groups are reported in Table 4.

**Table 4:** Methods of prevention of medication errors by nurses and nursing students in ZahedanUniversity of Medical Sciences in 2015

Nurse’s view point	
reduce working pressure by increasing the number of staff proportional to the number and condition of patients	%16/98
Education and improve nurses’ knowledge about drugs and proper medicine prescribing and medication with principles and techniques	%13/91
Availability of the necessary information about drugs, side effects and interactions in the wards	%70/84
Using infusion pumps in wards in order to avoid rapid infusion of dangerous drugs	%59/78
Improve the working environment such as lighting, temperature, humidity, noise, controlling the number of patients, the movement of the patient accompanying	%94/70
Inform and educate nurses about new drugs	%52/64
Choosing nurses for different wards according to their interests	%46/61
Paying attention to medication error reports as an opportunity to learn in order to prevent their recurrence	%18/57

**Nursing student’s view point**	

create a section as medication calculation to practice and improve the skills needed for calculating right dosage of the drugs	%77/96
Availability of pharmacological books and access to sites related to pharmacological information in the wards and holding periodical pharmacological congresses	%54/93
Awareness on the correct principles of giving drug, such as identifying the correct patient, correct drug, correct dosage, correct time and routine of the ward	%64/80
The use of electronic medical cards for the correct reading of medication orders by students	%12/66
Positive reaction of nurse educators toward reporting medication errors for better management of errors	%45/56

## 4. Discussion

The results of this study have shown that the most important factors that can be effective on the medication errors in nurses are: fatigue due to high workload, the large number of critically ill patients, doctor’s damaged and unreadable orders and the low nurse: patient ratio. In Hosseinzade’s et al. (2012) showed the most important causes of medication errors in nurses were the staff deficiency, fatigue due to high workload and high workload in the wards (Hosseinzadeh & Aghajari, 2012). In a study conducted by Blendon et al., low number of staff was mentioned as the leading cause of medical errors (Blendon & DesRoche, 2002). Also Tang’s study showed that, the low number of staff reduced the quality of work and increased the medication errors (Tang & Sheu, 2007). In this study fatigue due to high workload was reported as the first cause of medication errors. But in NikPeyma’s study, physical or mental fatigue was reported as the third cause of medication errors (Nikpeyma & Gholamnejad, 2009). However, according to studies, fatigue due to high workload is one of the main causes of medication errors. Study results had shown that some medication errors such as fast injection of drugs that must be injected slowly and not paying attention to the drugs that need much more attention than others are more common among nurses (Yaghoobi et al., 2015). As in the study of Howe et al. (2005) about a number of most common medication errors showed that, fatigue due to high workload is the most important cause of medication errors (Haw & Dickens, 2005). Exhaustion emerges as the sense of pressure specifically chronic f from high load working (Arbabisarjou et al., 2015). Findings of a study reported fatigue as the third cause of medication errors. They believed that long working hours and high workload and increase in environmental stimulations such as noise and inappropriate lightening in working environment can lead to medication errors. They believe that multiple and complex roles and functions that are simultaneously expected from nurses can increase the incidence of medication errors in them (Pape & Guerra, 2005). In another study, the most important cause of medical errors was related to nurses’ high workload and their unfamiliarity with the patient’s condition had less impact among the effective factors of medication errors (Al-Shara, 2011). According to the study results it became clear that many factors are involved in medication errors, and also human errors are inevitable (Wolf & Hicks, 2006). But proper planning and a monitoring and caring system can reduce the errors and prevent the dangerous results of errors in time of occurrence. Medication errors lead to distrust of the patient and his family toward the healthcare system and also lead to increasing the costs that this problem relates to different causes such as lack of awareness and knowledge and not paying attention to the drug prescription standards (American Society of Hospital Pharmacists 1993). Study results stated that false medicinal calculations, insufficient pharmacological knowledge and Illegibility of patients’ records are the most common medication errors among students. (Gorgich et al., 2014) About the nurses’ view point on how to prevent medication errors the results showed that reducing the work pressure by increasing the number of staff proportional to the number and status of patients, is the most important strategy to avoid these error. While in another study in this field increasing the number of staff proportional to the number of patients, staff training and information about new drugs were the most important ways to prevent medication errors (Ghasemi & Valizadeh, 2009). The study showed that the main causes of medication errors in nursing students were: wrong medication calculations, lack of pharmacological information, unreadable orders in medicine cards, environmental conditions lead to distraction and having stress in the emergency situation, that they were reported as five causes that have most effect on medication error occurrence in nursing students. In Esmaeil nejad’s study entering wrong drug in the medicine card and not paying attention to the dosage of the drug in that were reported as causes of medication errors. However the highest rate of medication errors occurred in the emergency ward (Nejad & Hojjati, 2010). In the study of Wolf and colleagues the most common causes of medication errors in students were poor clinical performance, not following-up the treatment methods and lack of pharmacological information in students (Wolf & Hicks, 2006). In a study conducted by Kouhestani as the amount, type and causes of medication errors in nursing students, lack of pharmacological information, not paying attention to the amount of drug in the medicine card and wrong medication calculation were reported as most common causes of medication errors occurrence (Koohestani & Baghcheghi, 2008), that is completely compatible with the results of this study about the causes of medication errors in students. They concluded that more familiarity with drug information at school and clinical internship can be effective in reducing medication errors occurrence. Health care providers need to identify the causes of errors to find their solutions and reduce the number of them and get better results for improving the situation. Effective pharmacological management is a nursing task that links the scientific ability, technical skills and practices based on compliance (Soozani & Bagheri, 2007).

## 5. Conclusion

Finally, with regard to the causes of medication errors and the importance of them, which are a measure of the quality of health care and due to increasing number of critically ill patients in wards that have a significant role in the incidence of medication errors it is necessary for the nursing managers to solve the lack of manpower proportional to number of patients. Also familiarity and education of nurses with the impressive processes in reducing medication errors and making electronic medicine cards for patients can lead to medication error reducing. To control and reduce risk factors of medication errors followings are recommended: having a systematic approach to recognize the effective causes of medication errors and trying to solve them, quantitative and qualitative increasing of the knowledge of nursing students about the medication errors that results in improving students function and reducing the medication errors, holding retraining periodical classes about pharmacological knowledge according to student’s needs, easy access to internet for students in health centers for updating their pharmacological information and students continuous evaluation about their pharmacological knowledge in the clinical internship course.
